# Supplementary material for: The right hippocampus leads the bilateral integration of gamma-parsed lateralized information
Source: eLife. 2016 Sep 6;5:e16658. doi: 10.7554/eLife.16658 (PMC5050016; doi:10.7554/eLife.16658)
Supplement: Figure 5—source data 2. — The data pertain to Figure 5B and Figure 5—figure supplement 2. DOI: http://dx.doi.org/10.7554/eLife.16658.016 [file elife-16658-fig5-data2.pptx]

## Slide 1
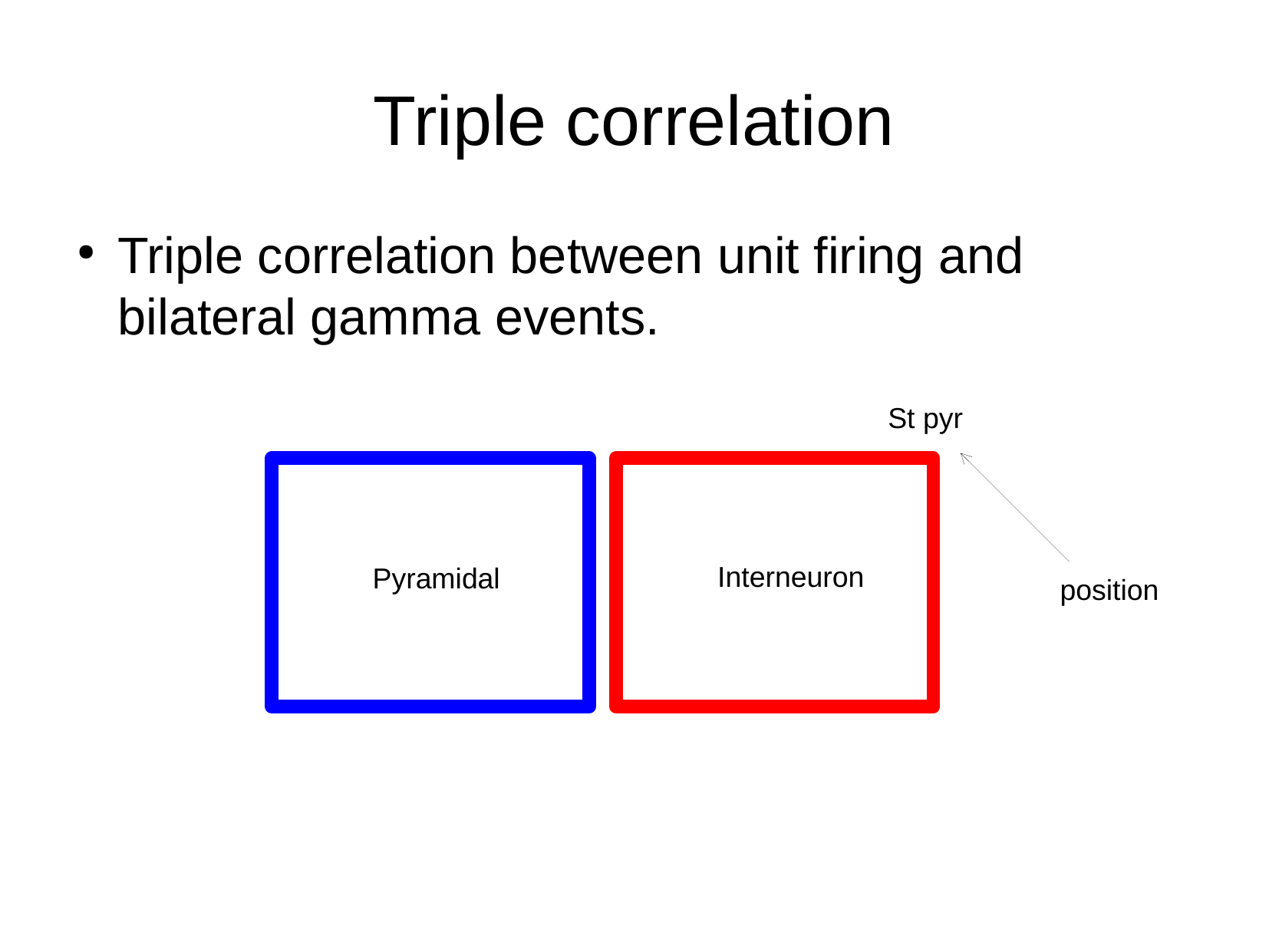

Triple correlation
Triple correlation between unit firing and bilateral gamma events.
St pyr
Interneuron
Pyramidal
position

## Slide 2
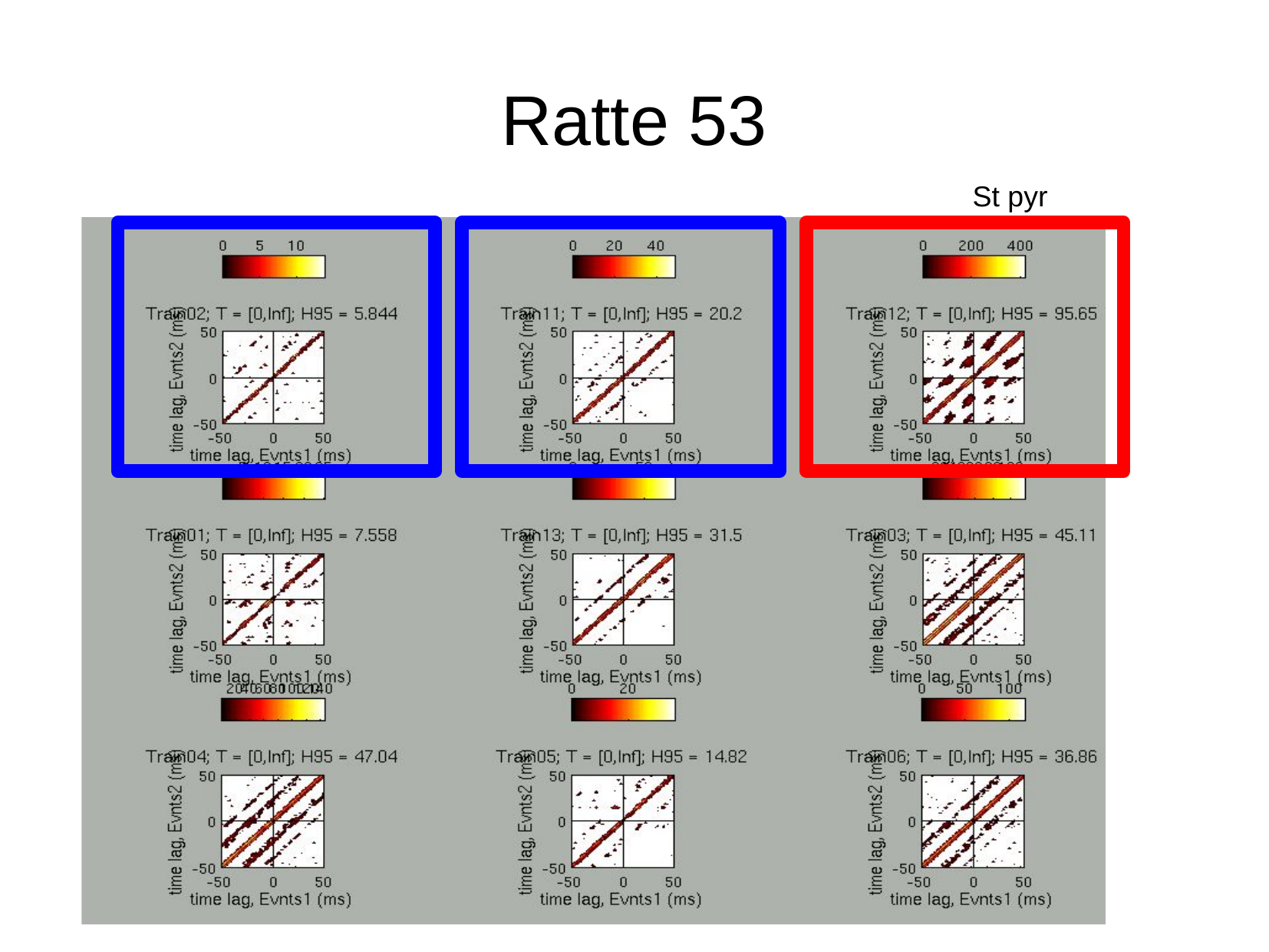

Ratte 53
St pyr

## Slide 3
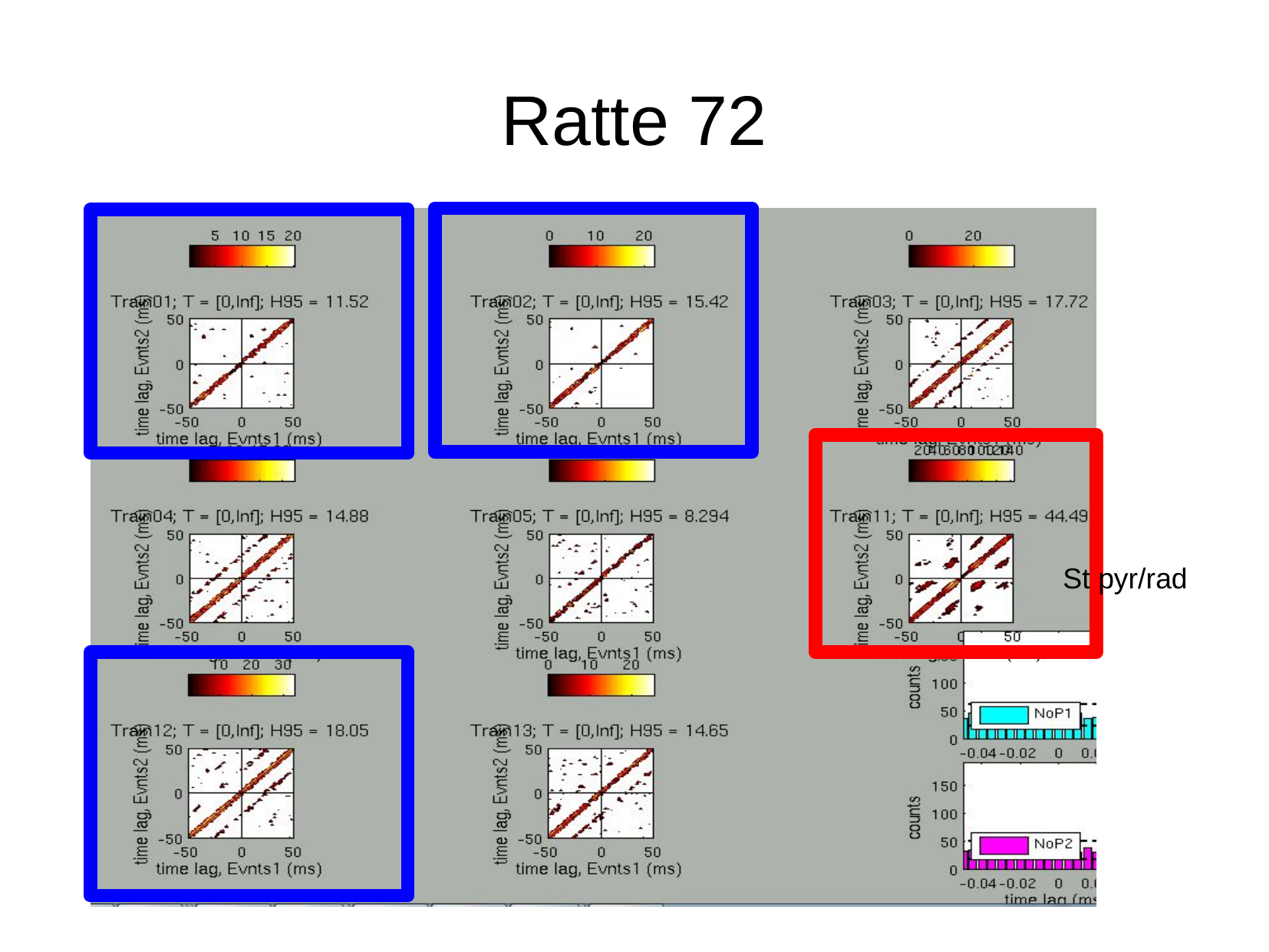

Ratte 72
St pyr/rad

## Slide 4
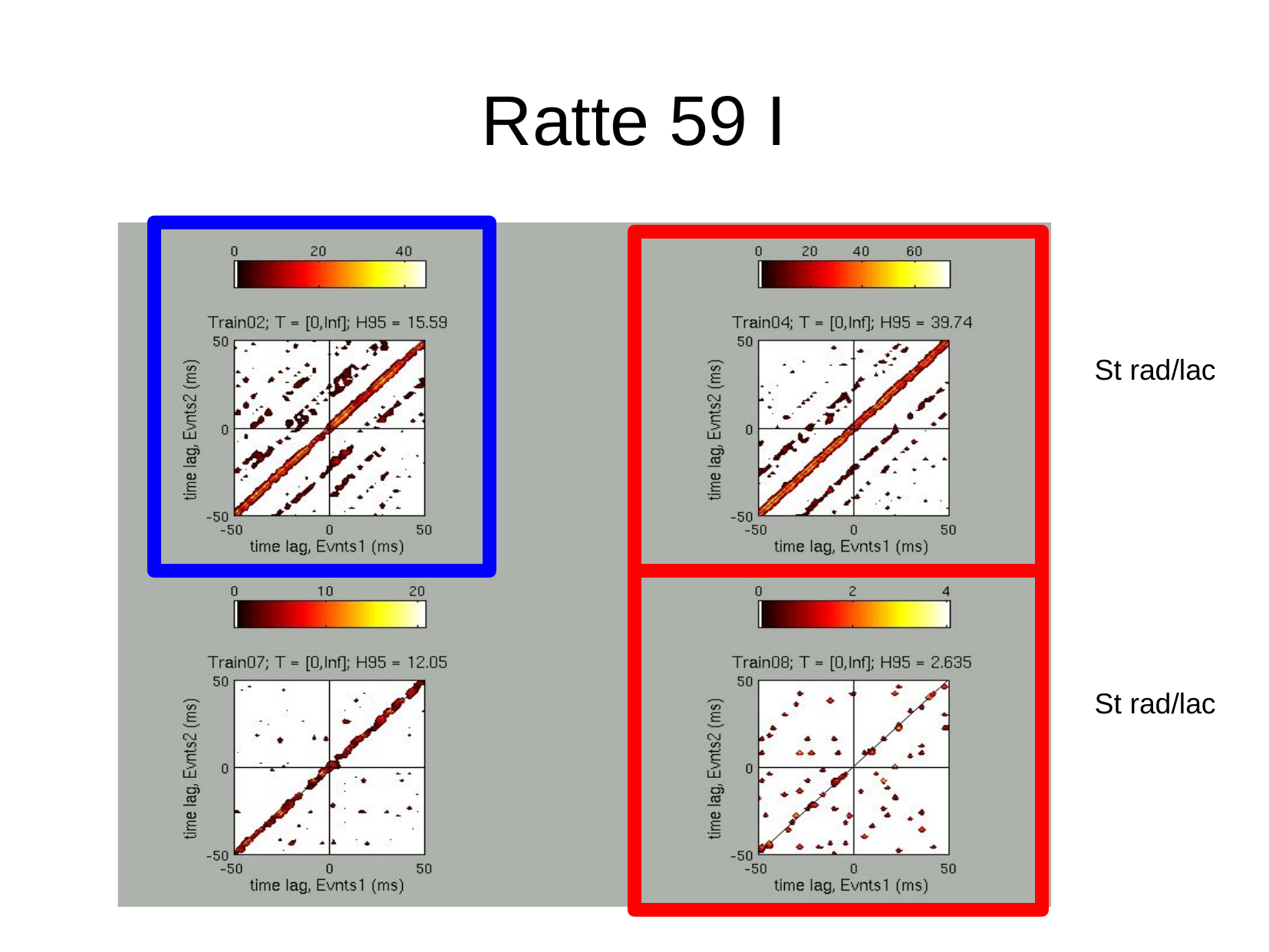

Ratte 59 I
St rad/lac
St rad/lac

## Slide 5
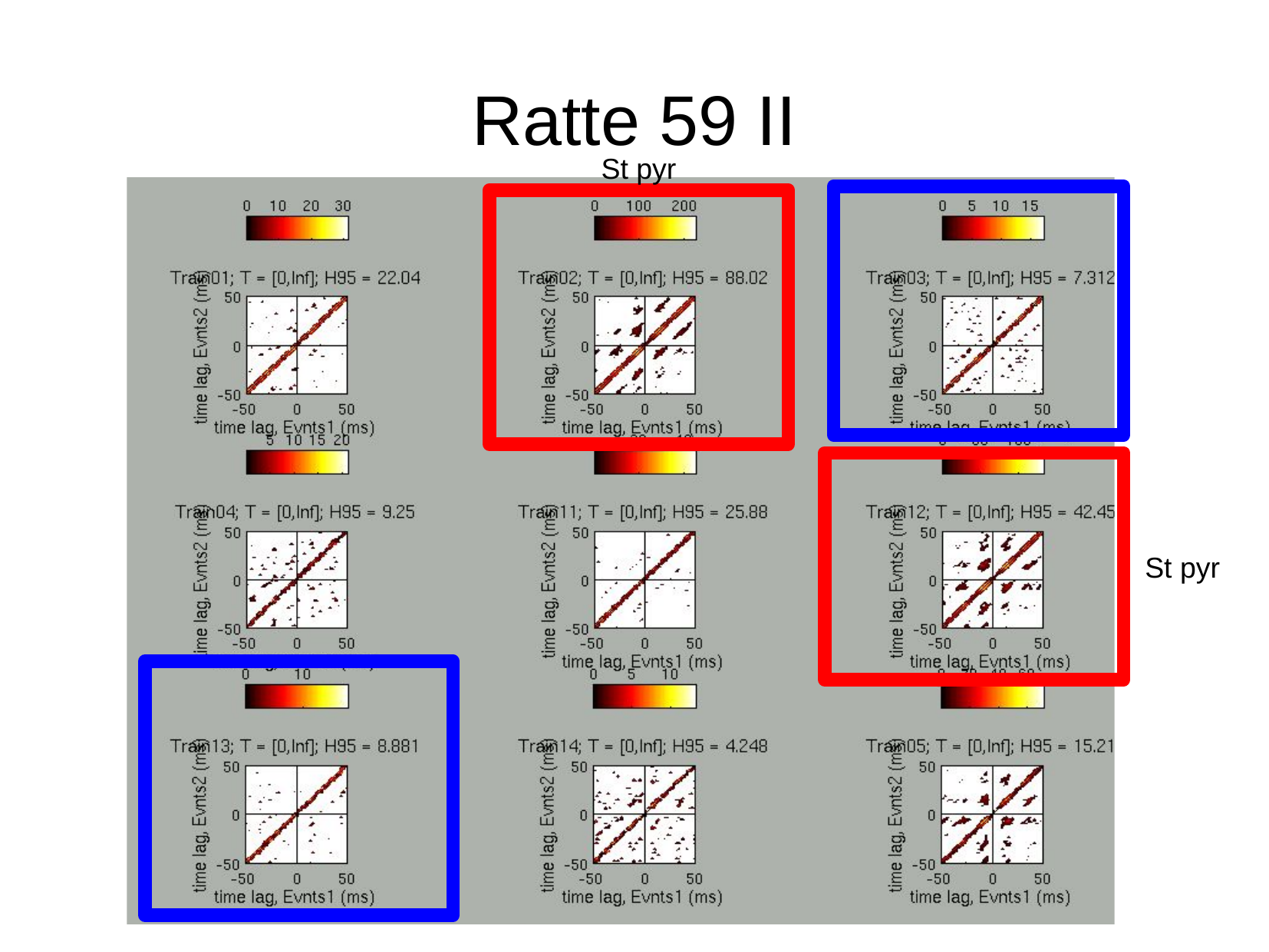

Ratte 59 II
St pyr
St pyr

## Slide 6
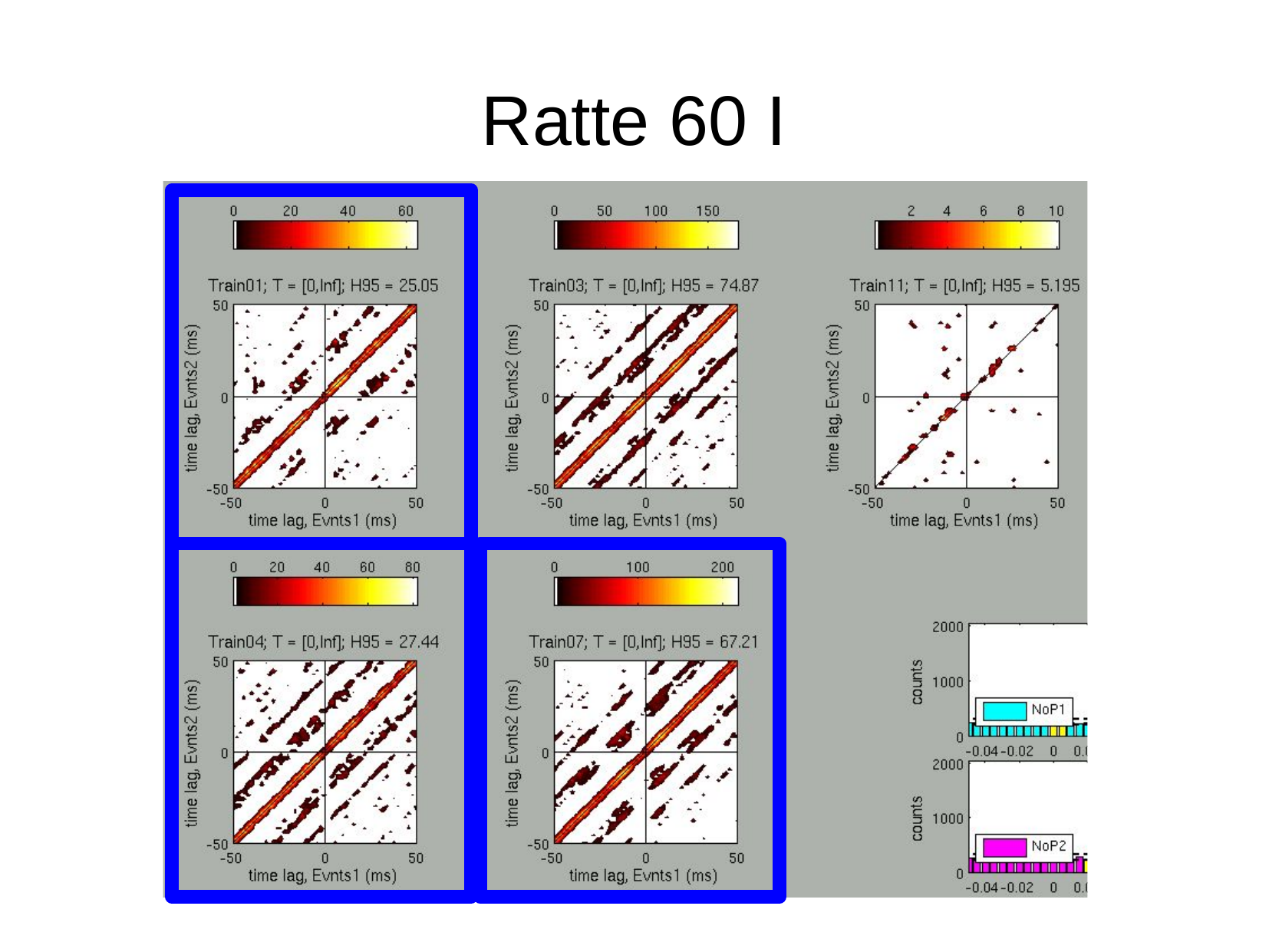

Ratte 60 I

## Slide 7
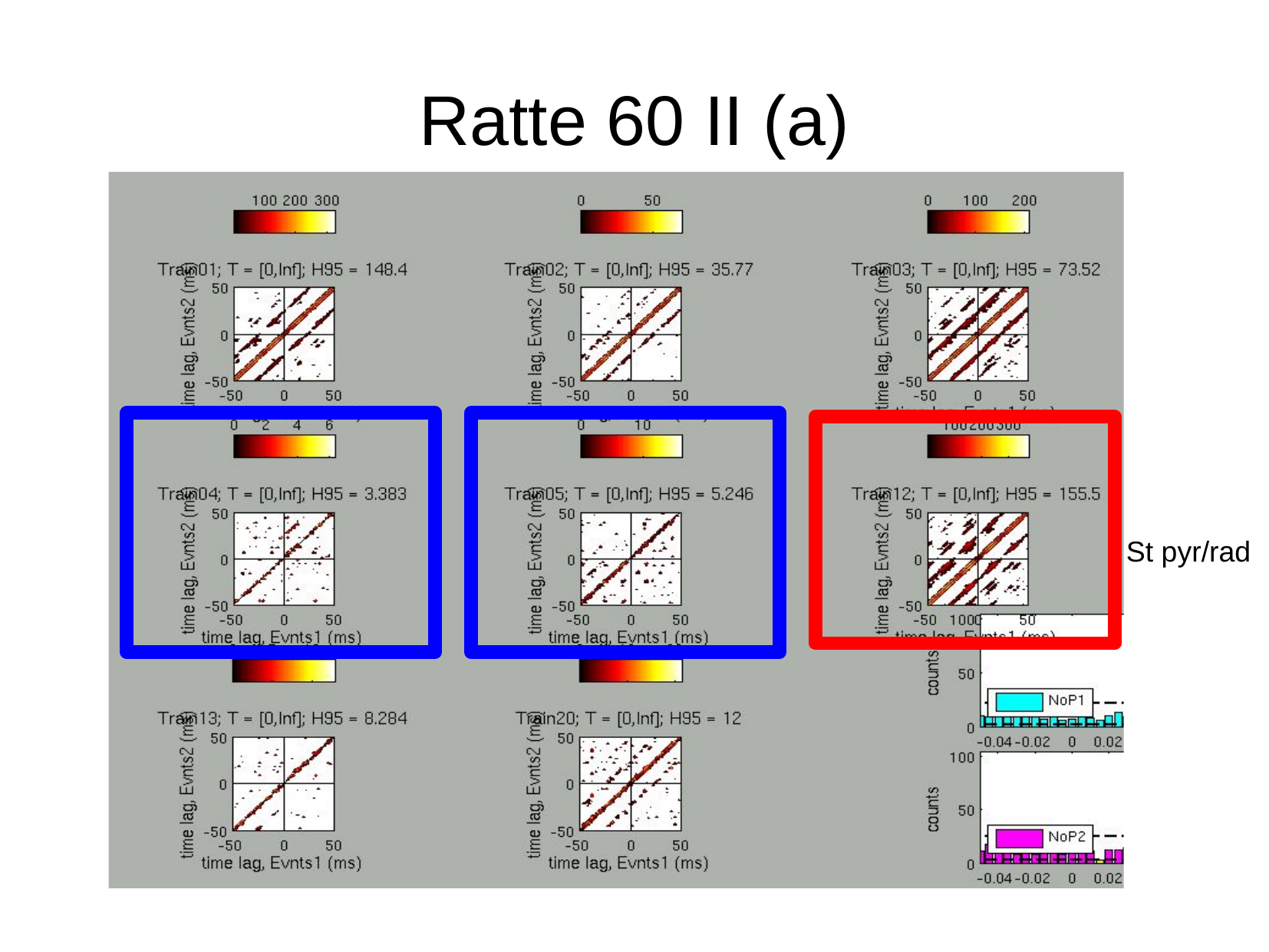

Ratte 60 II (a)
St pyr/rad

## Slide 8
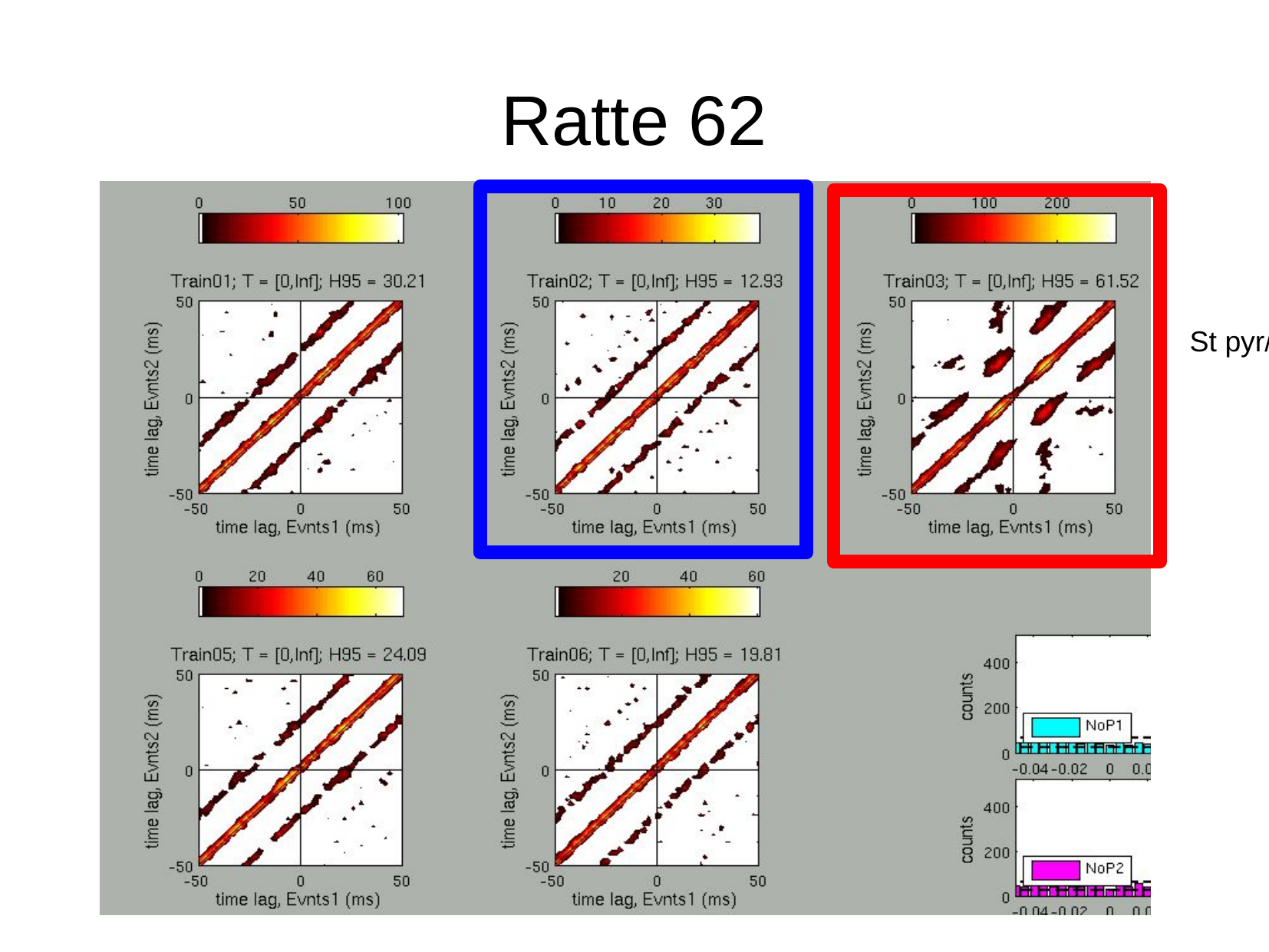

Ratte 62
St pyr/rad

## Slide 9
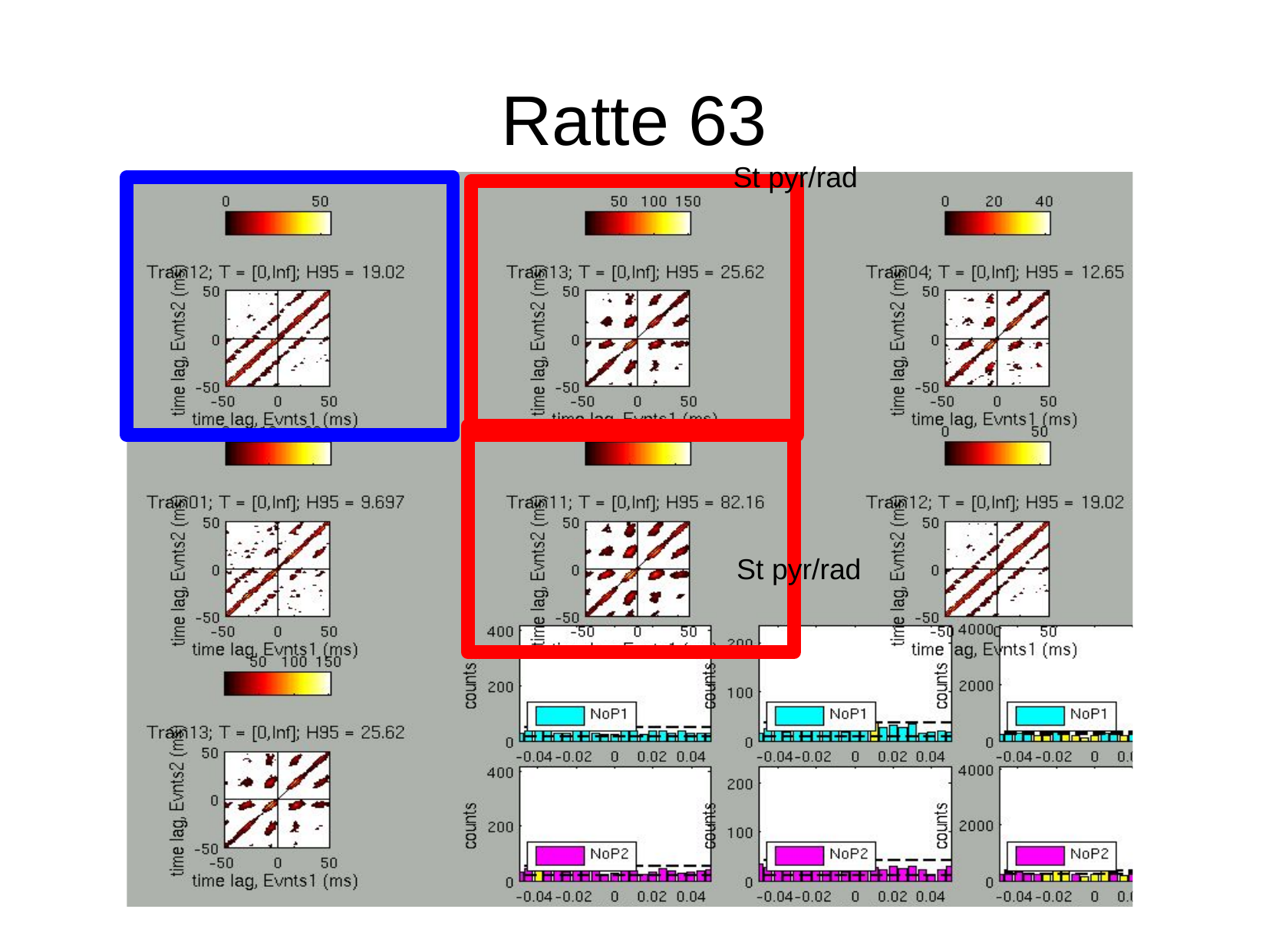

Ratte 63
St pyr/rad
St pyr/rad

## Slide 10
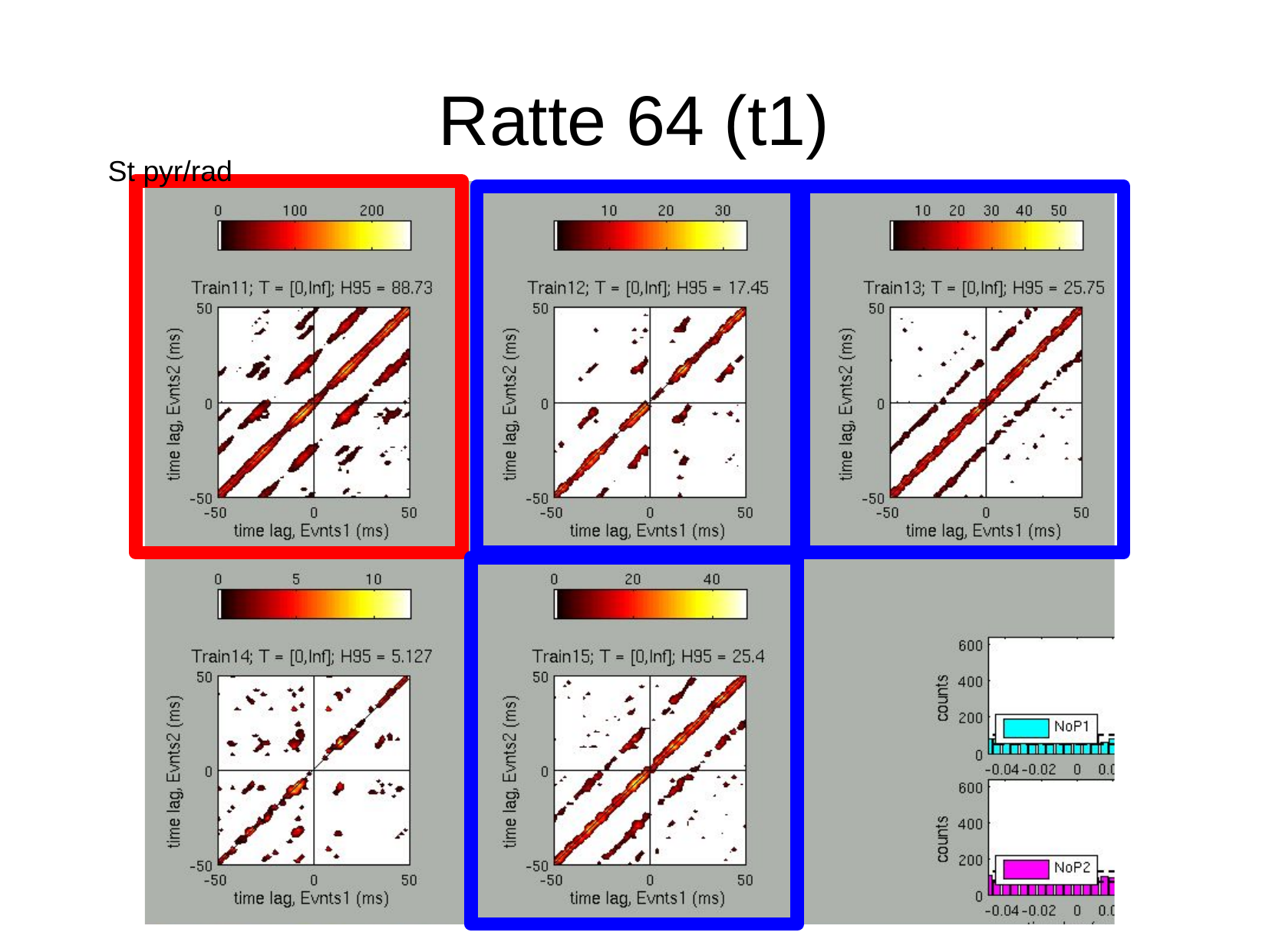

Ratte 64 (t1)
St pyr/rad

## Slide 11
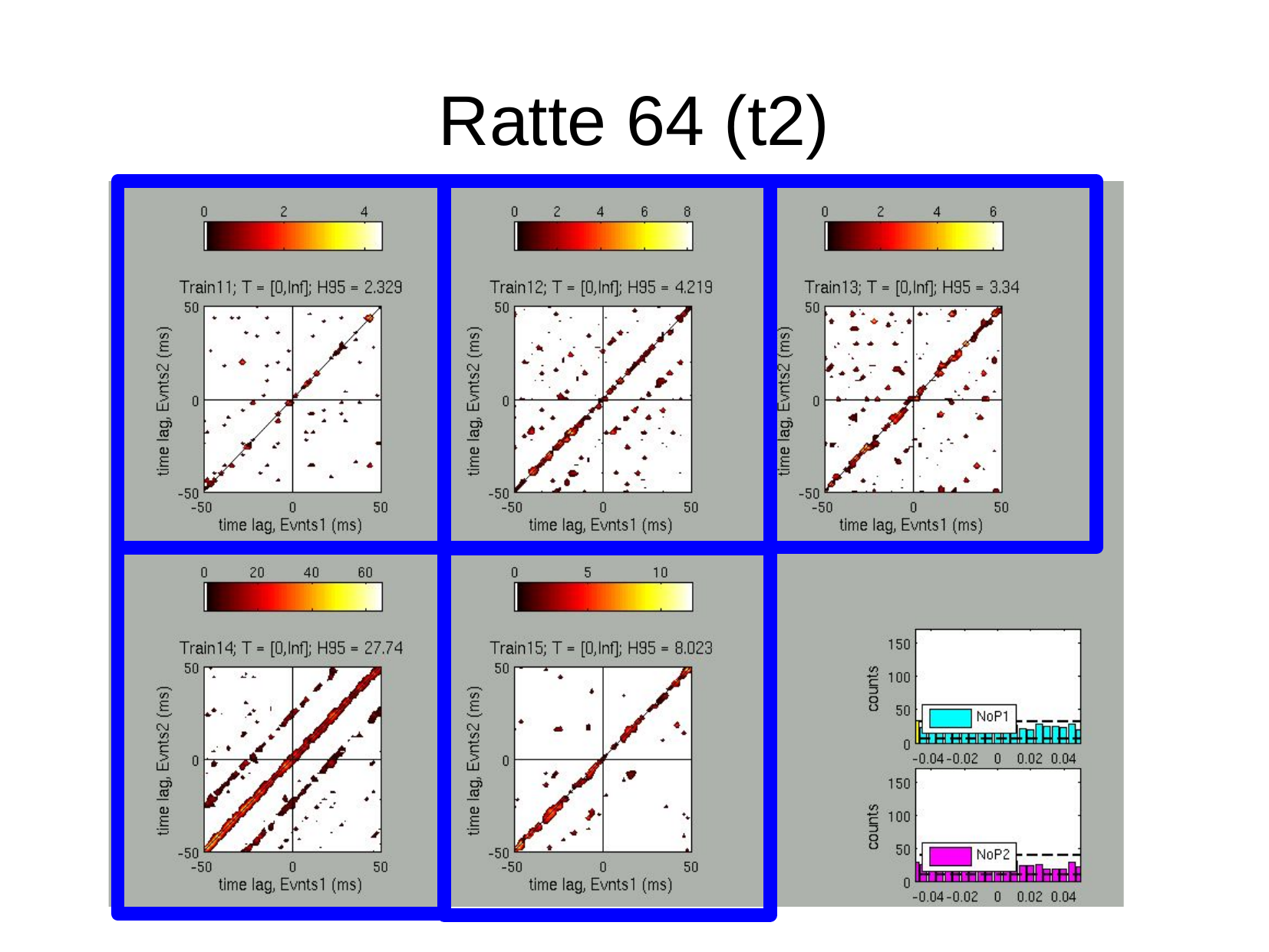

Ratte 64 (t2)

## Slide 12
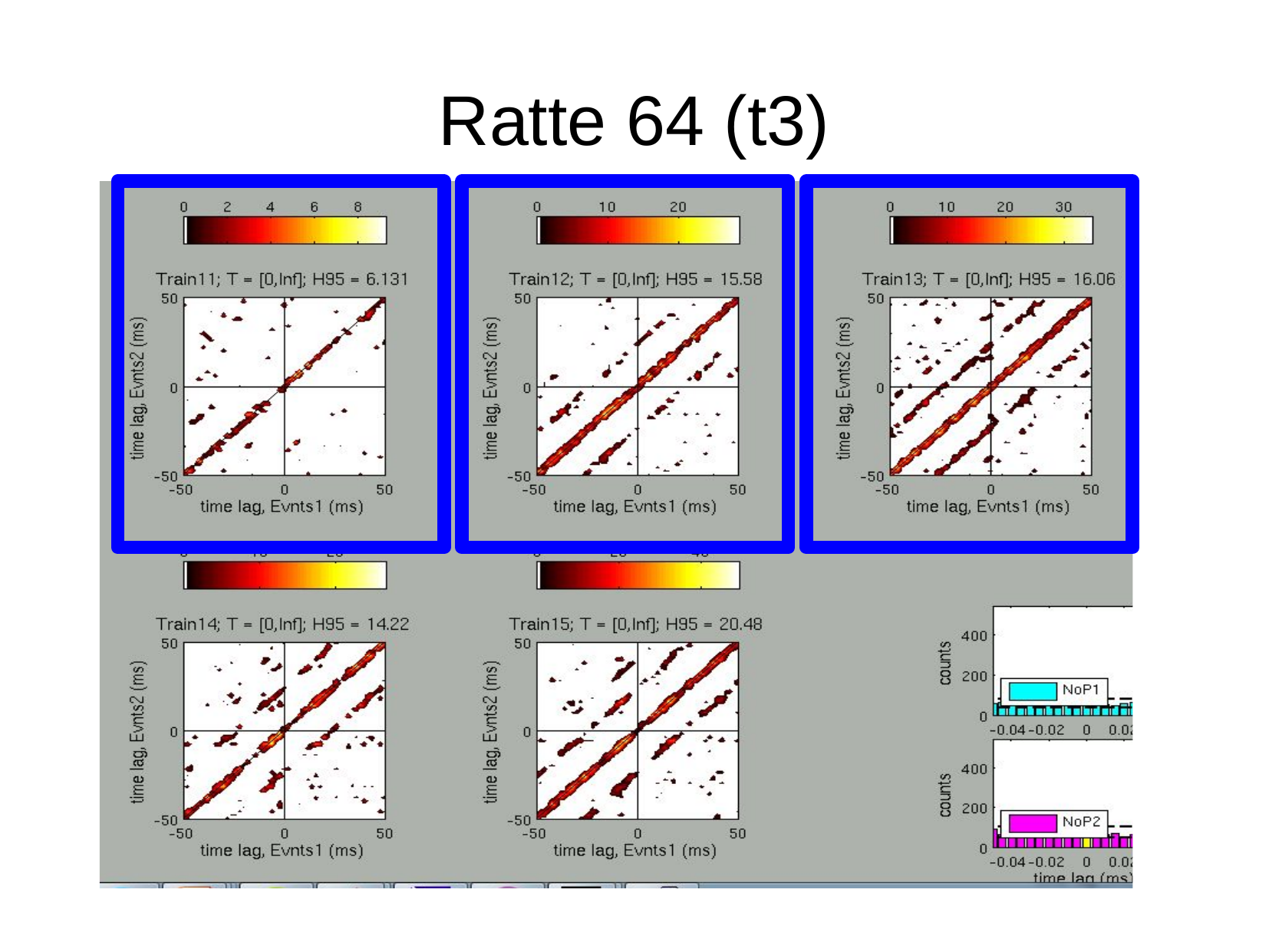

Ratte 64 (t3)

## Slide 13
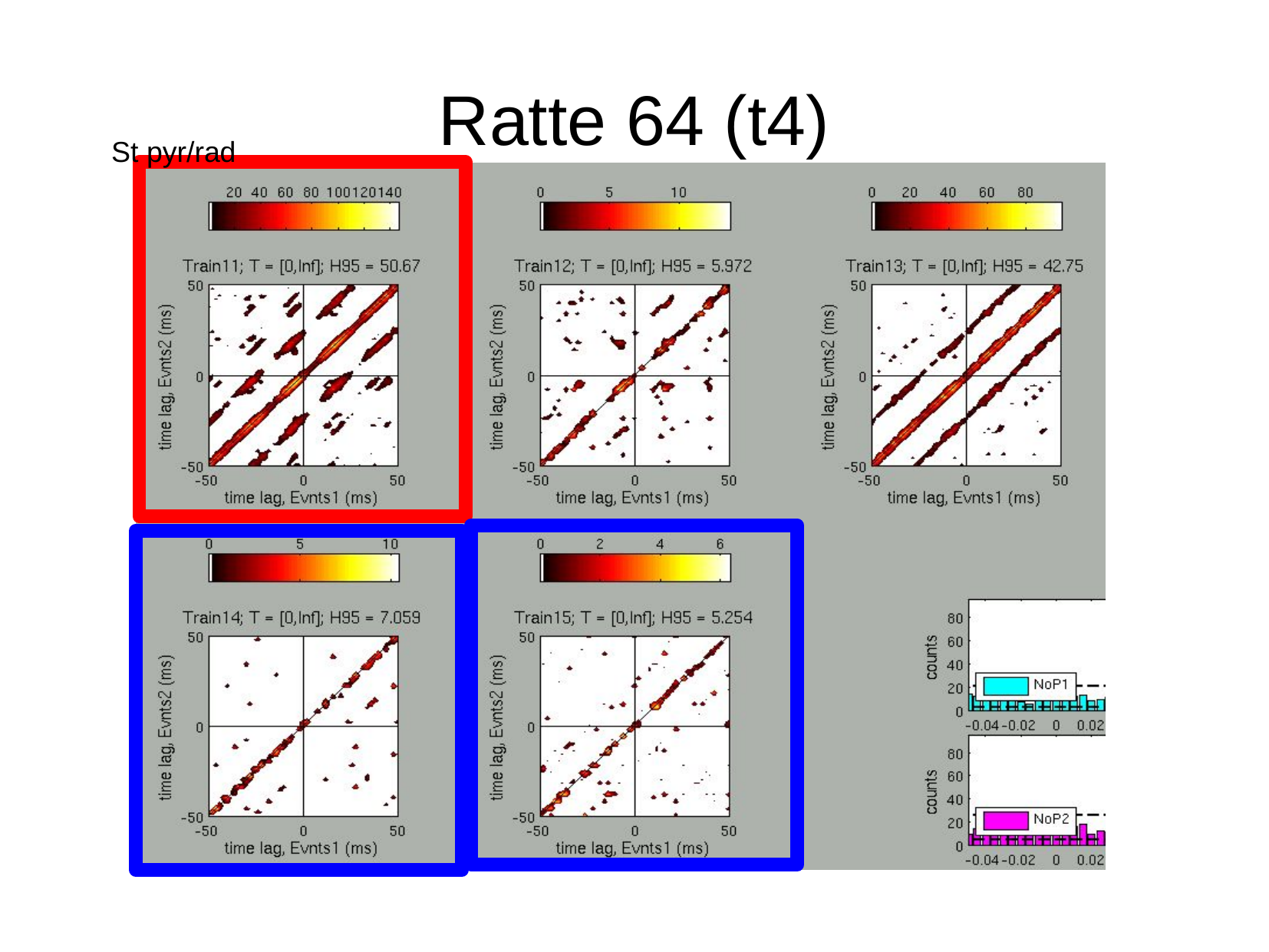

Ratte 64 (t4)
St pyr/rad

## Slide 14
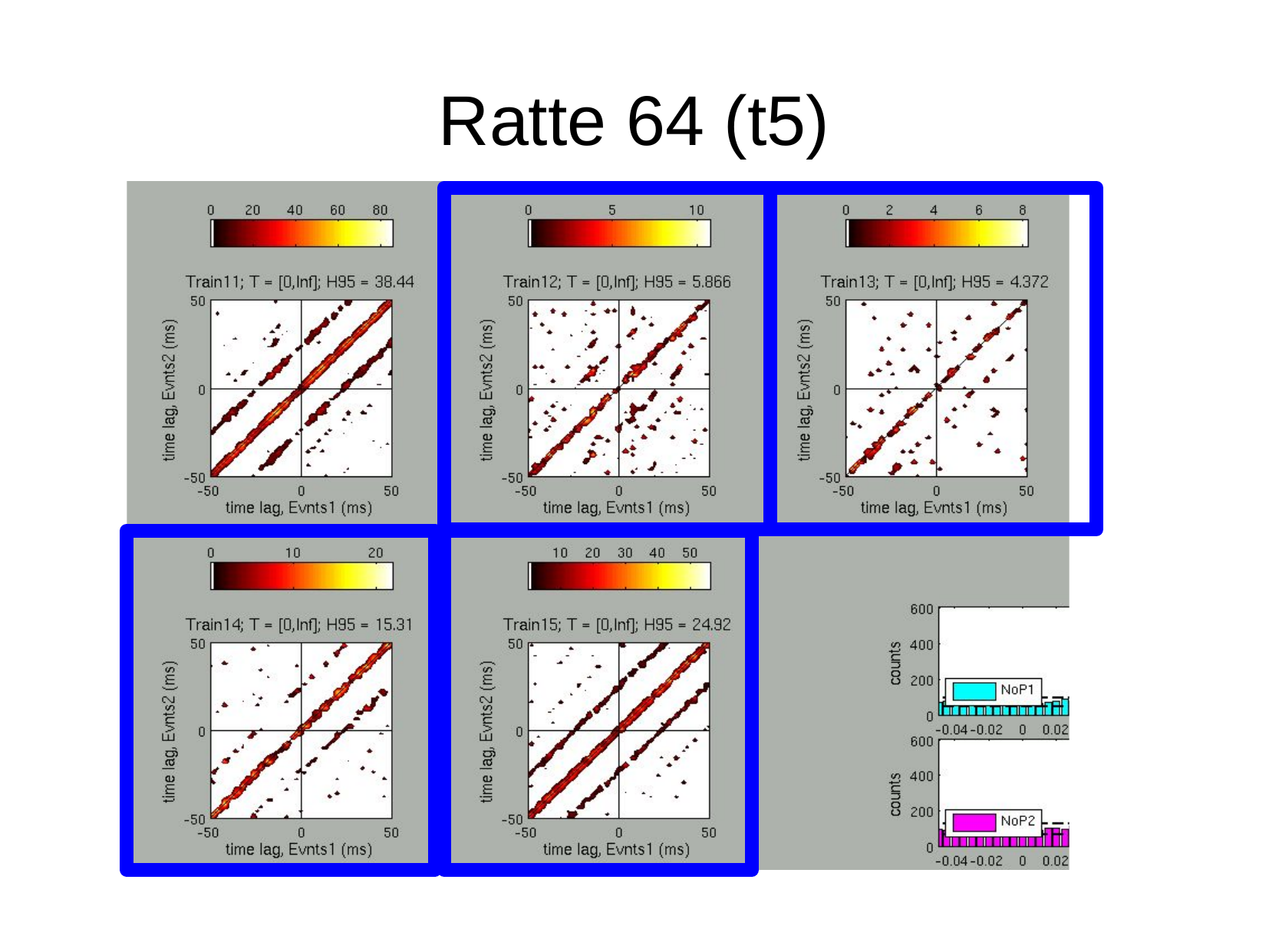

Ratte 64 (t5)

## Slide 15
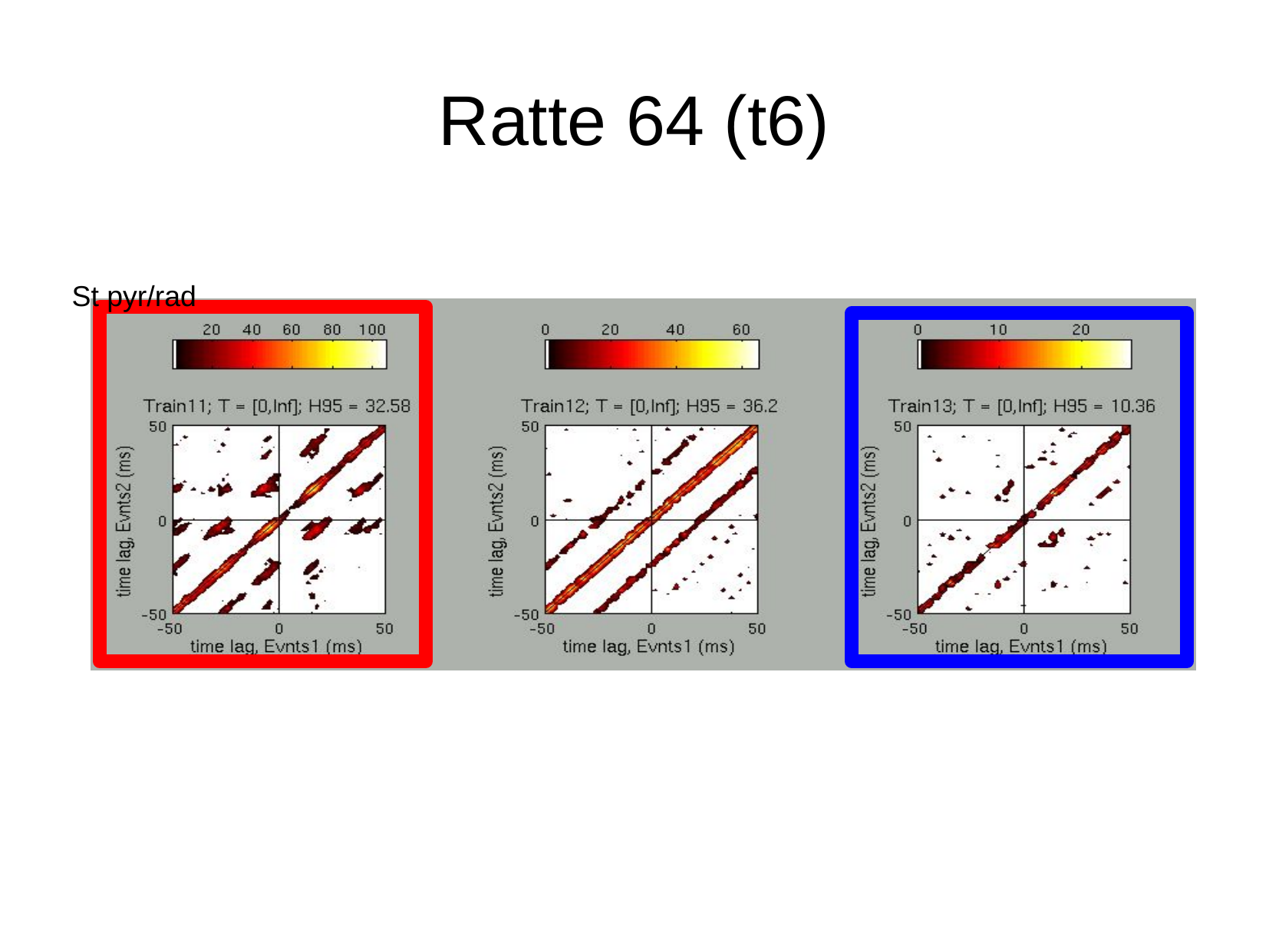

Ratte 64 (t6)
St pyr/rad

## Slide 16
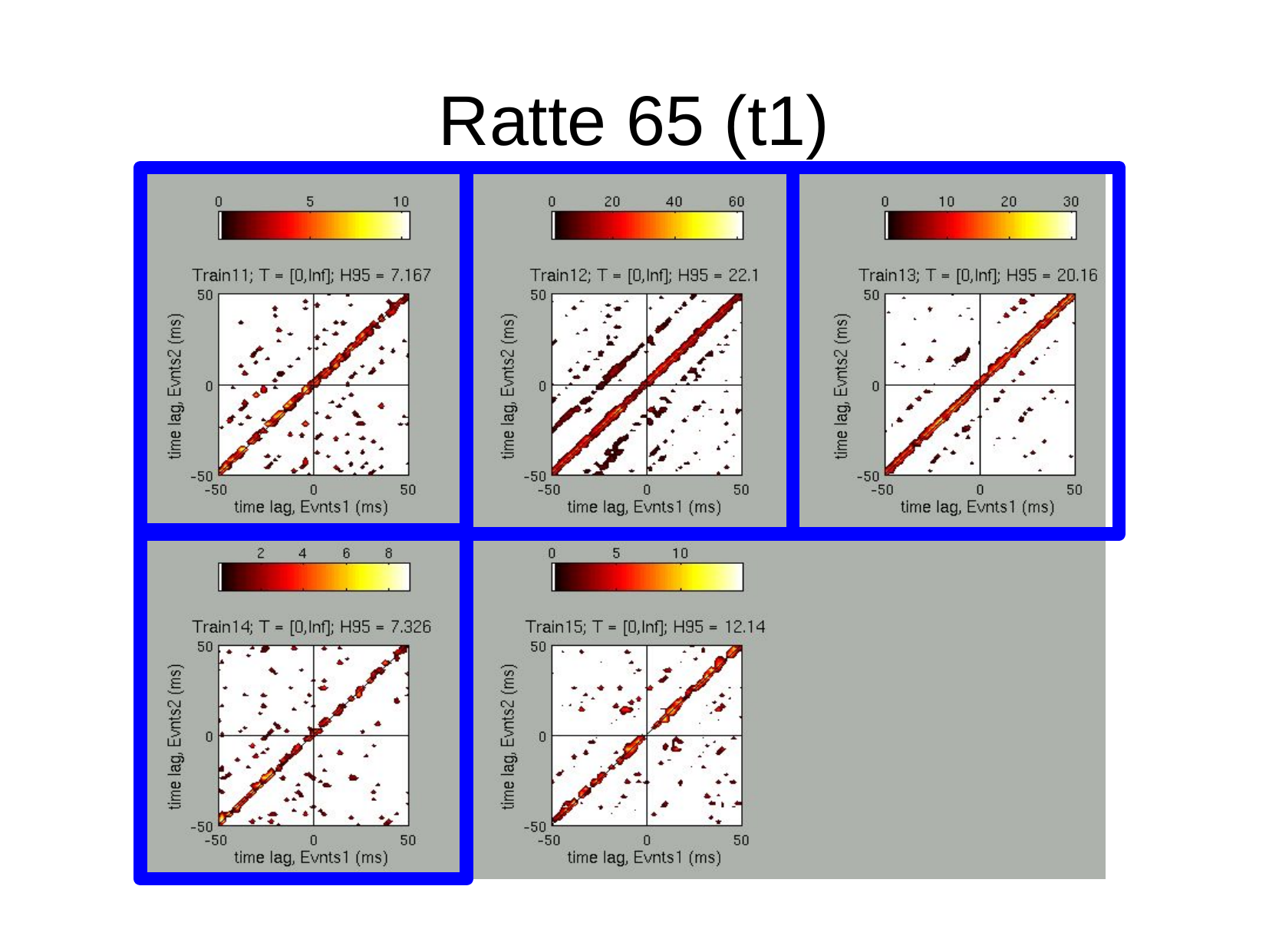

Ratte 65 (t1)

## Slide 17
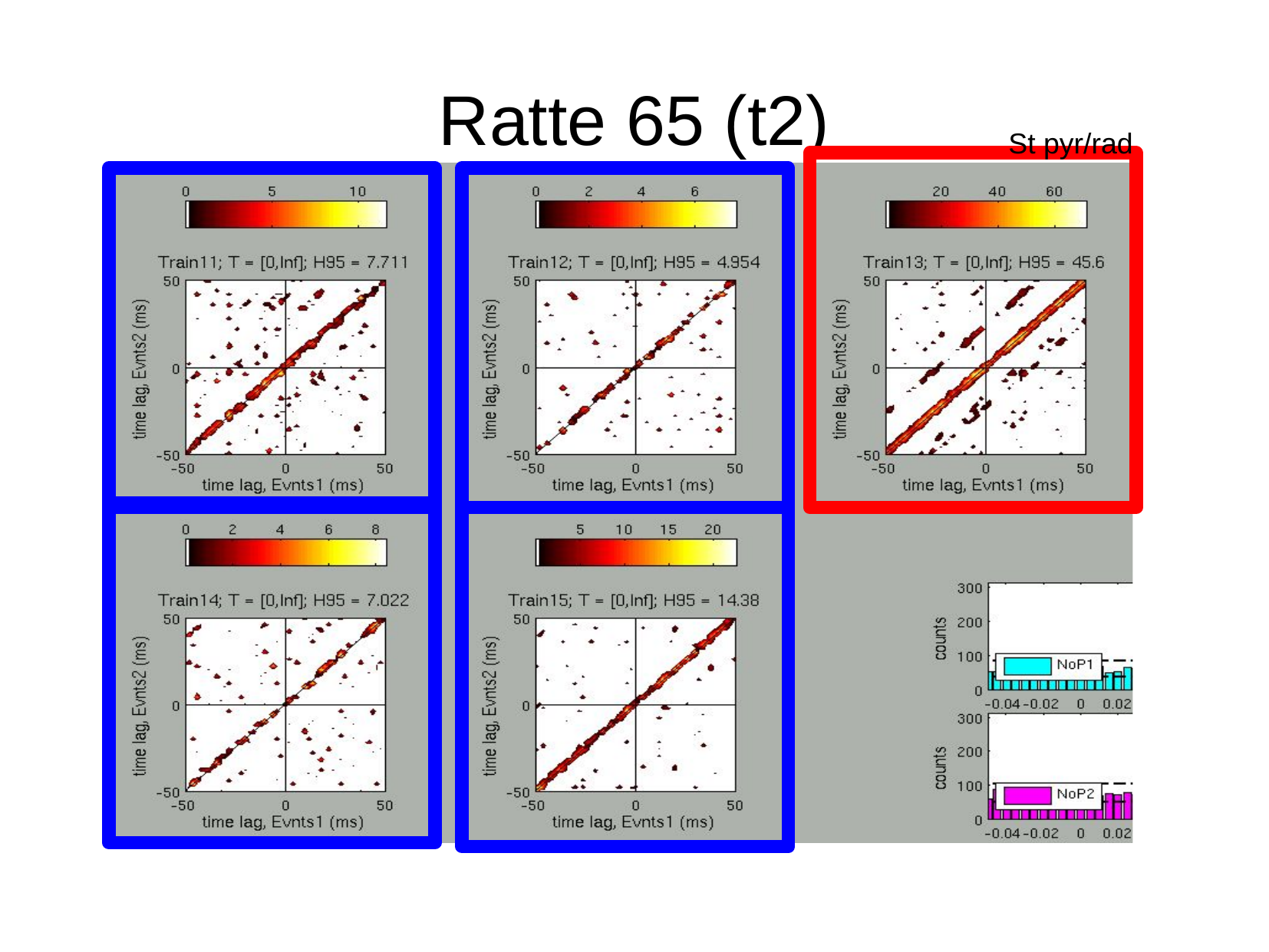

Ratte 65 (t2)
St pyr/rad

## Slide 18
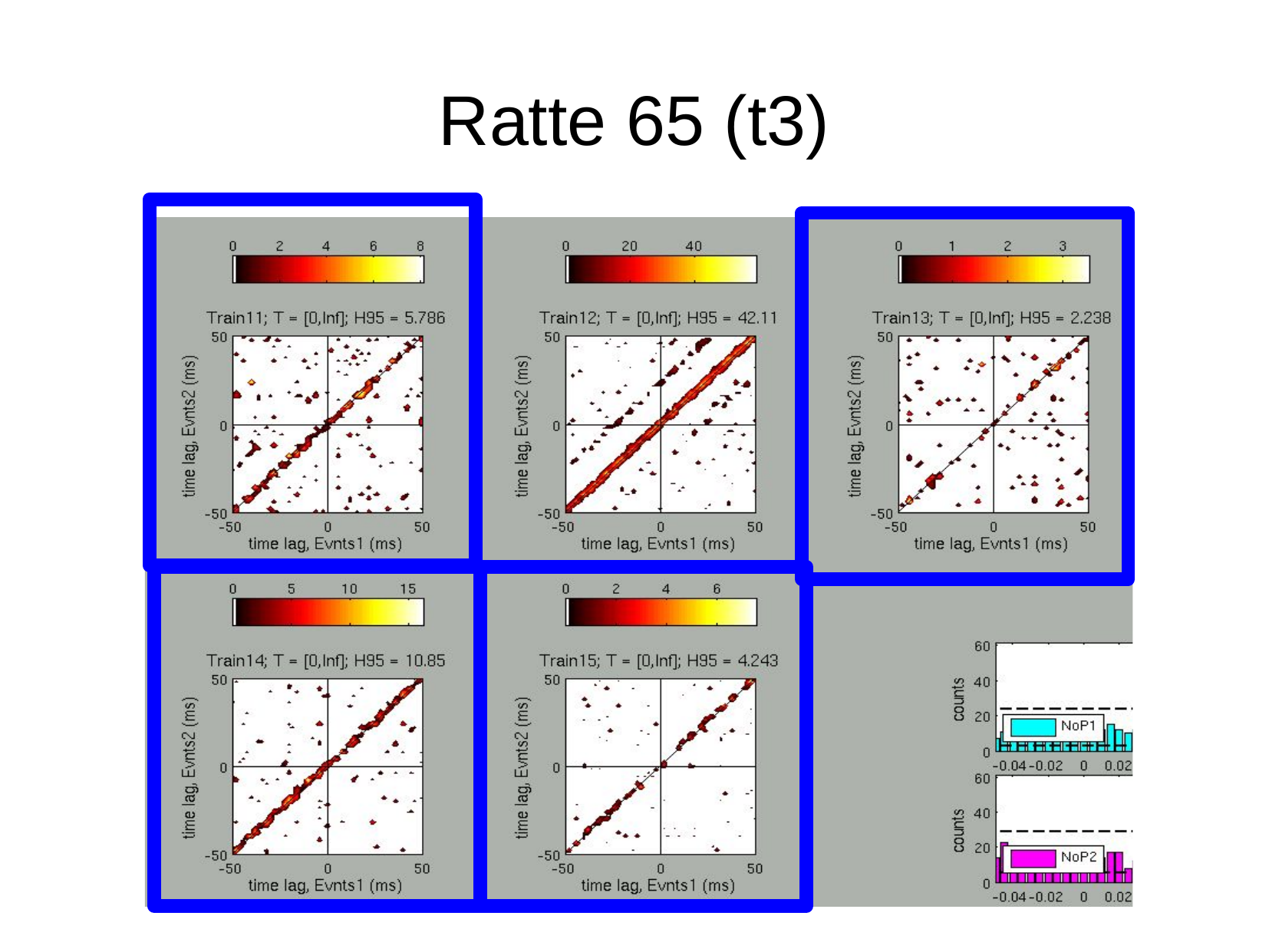

Ratte 65 (t3)

## Slide 19
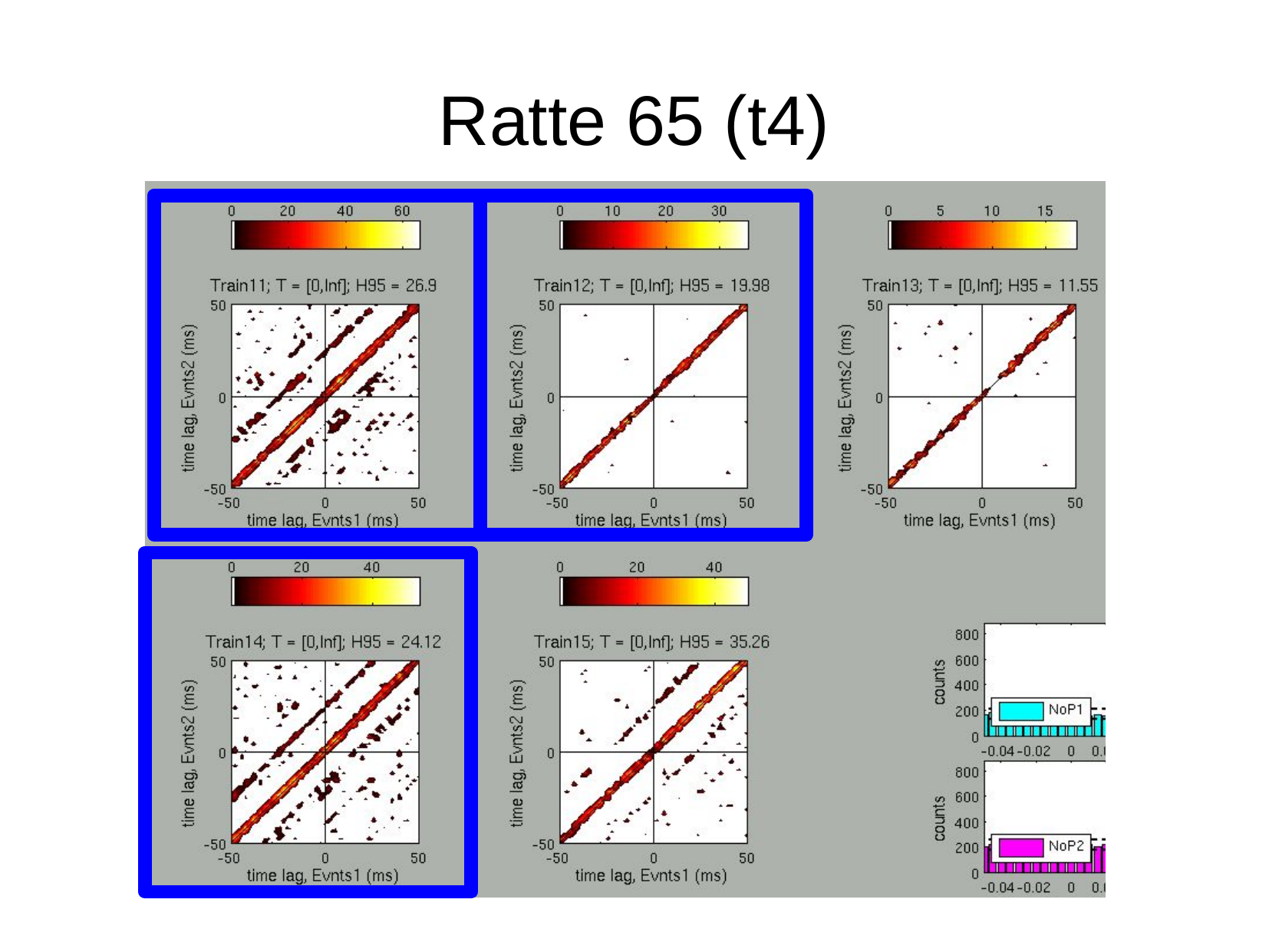

Ratte 65 (t4)

## Slide 20
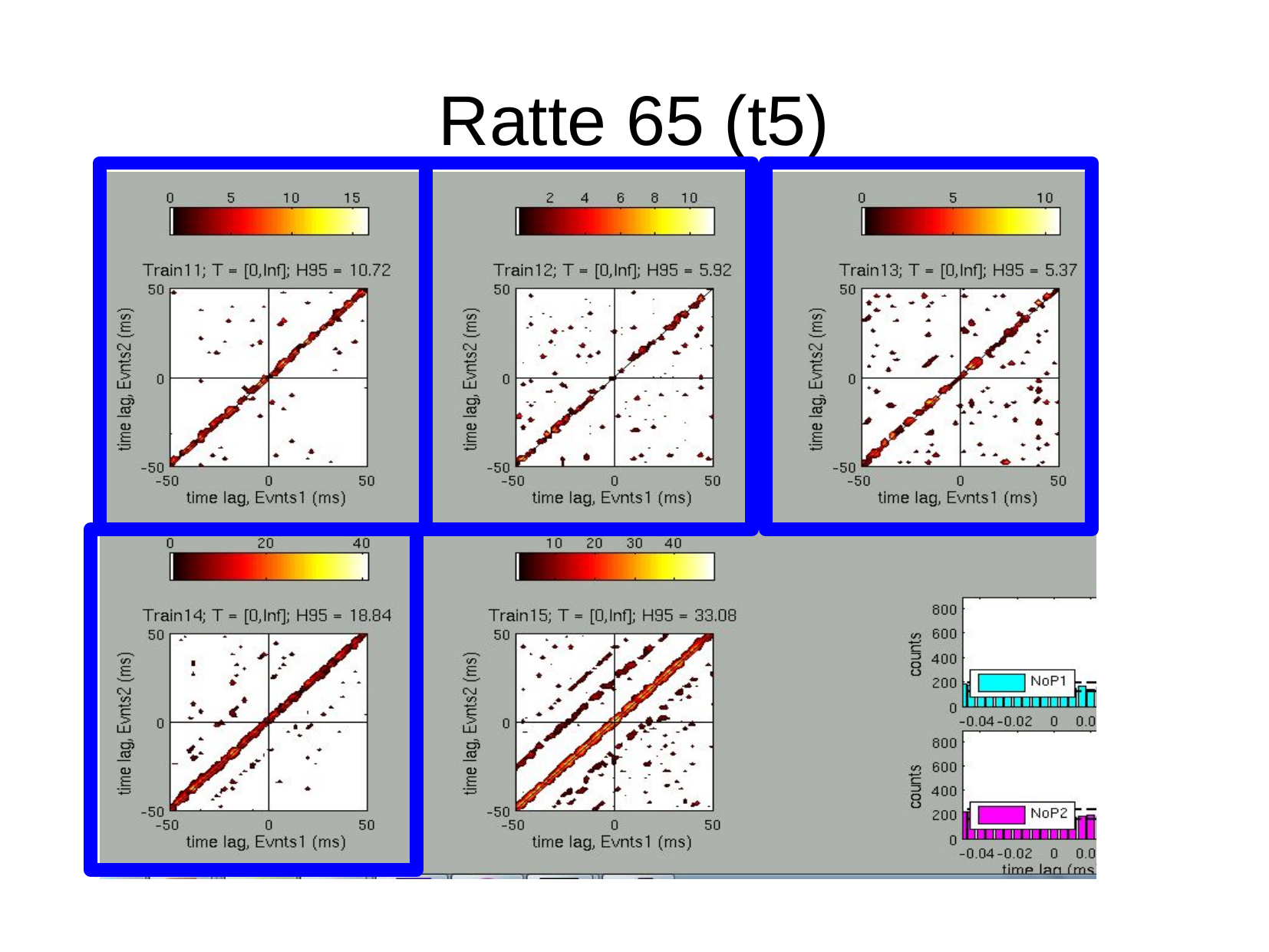

Ratte 65 (t5)

## Slide 21
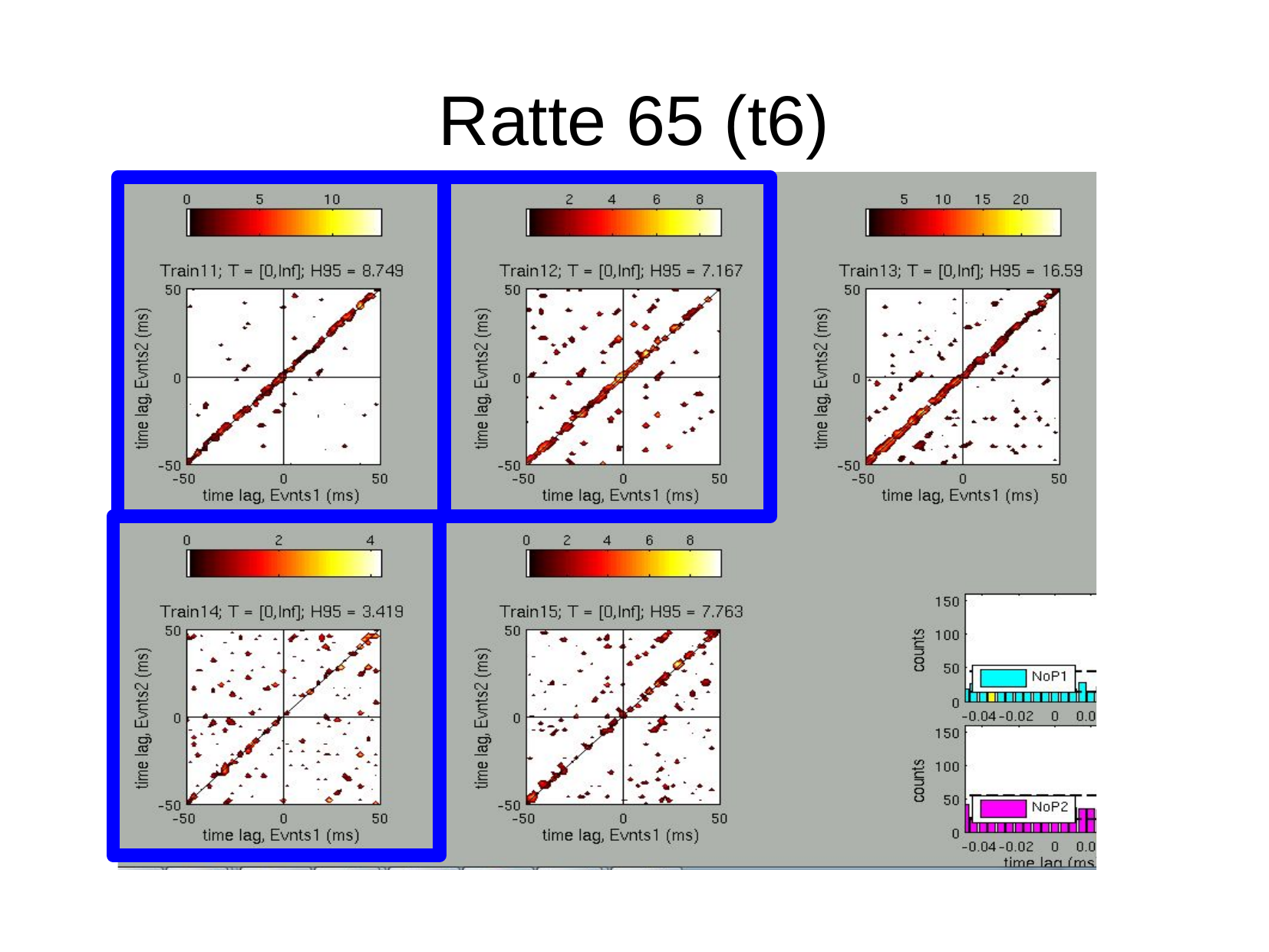

Ratte 65 (t6)

## Slide 22
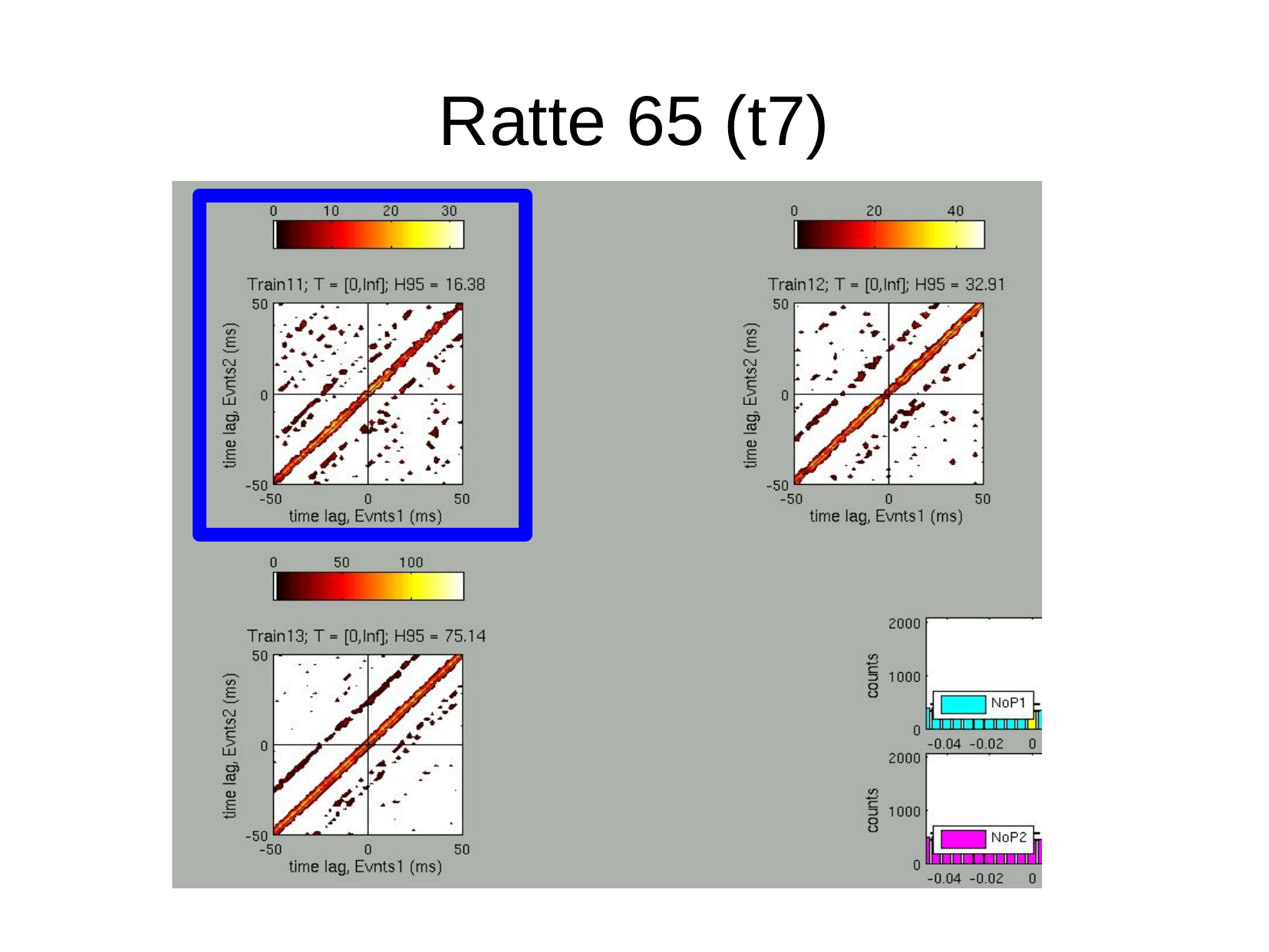

Ratte 65 (t7)

## Slide 23
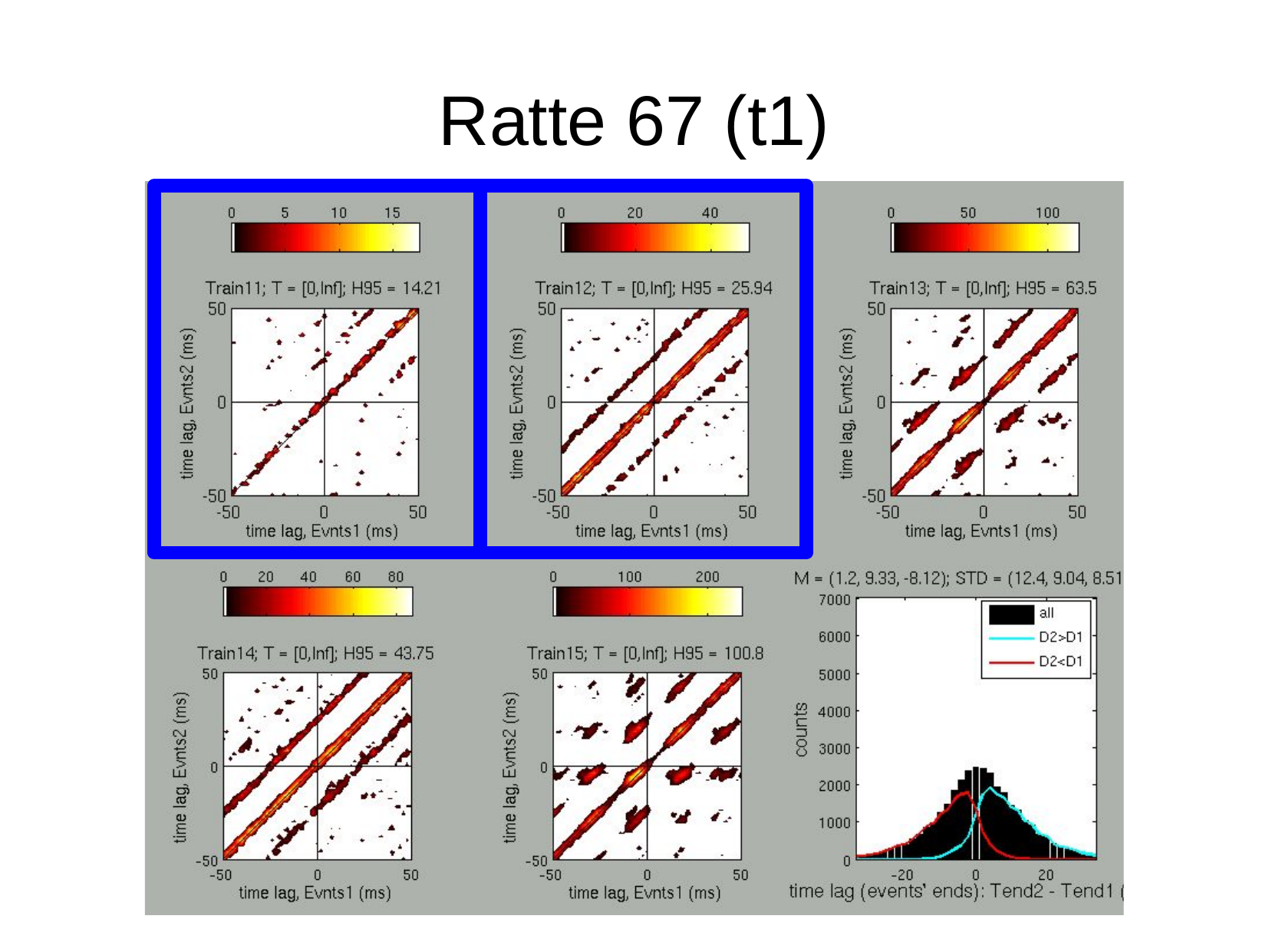

Ratte 67 (t1)

## Slide 24
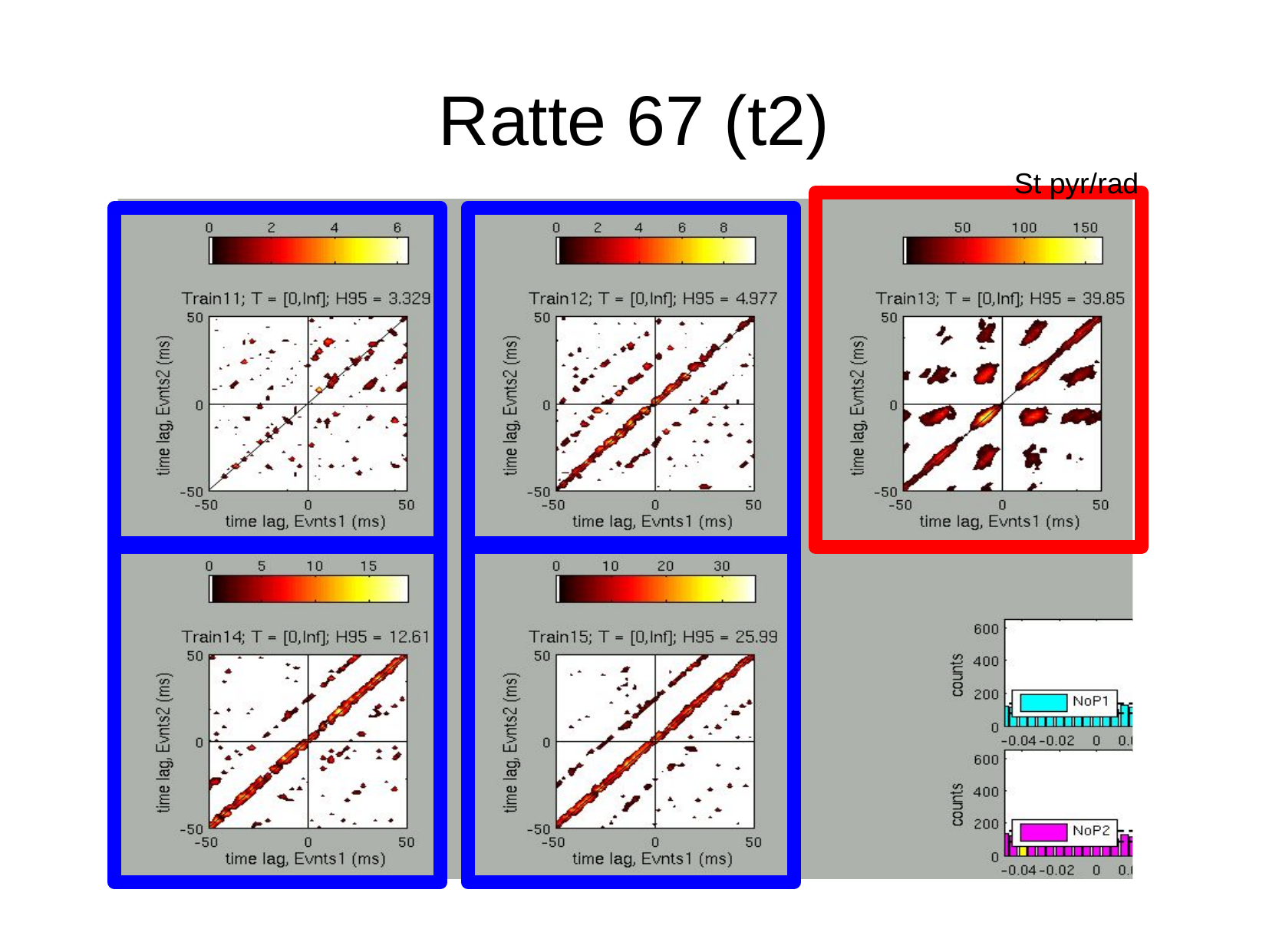

Ratte 67 (t2)
St pyr/rad

## Slide 25
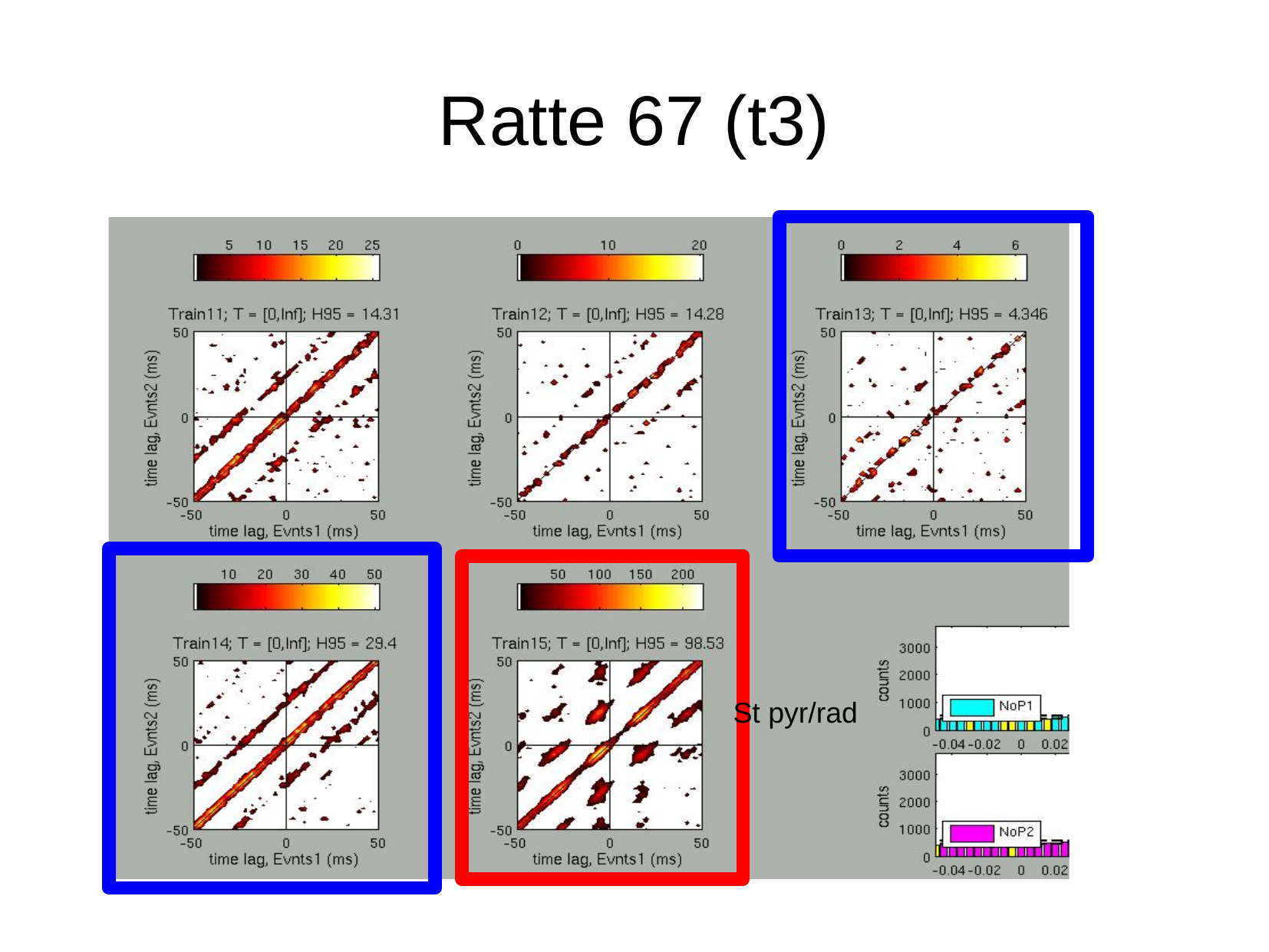

Ratte 67 (t3)
St pyr/rad

## Slide 26
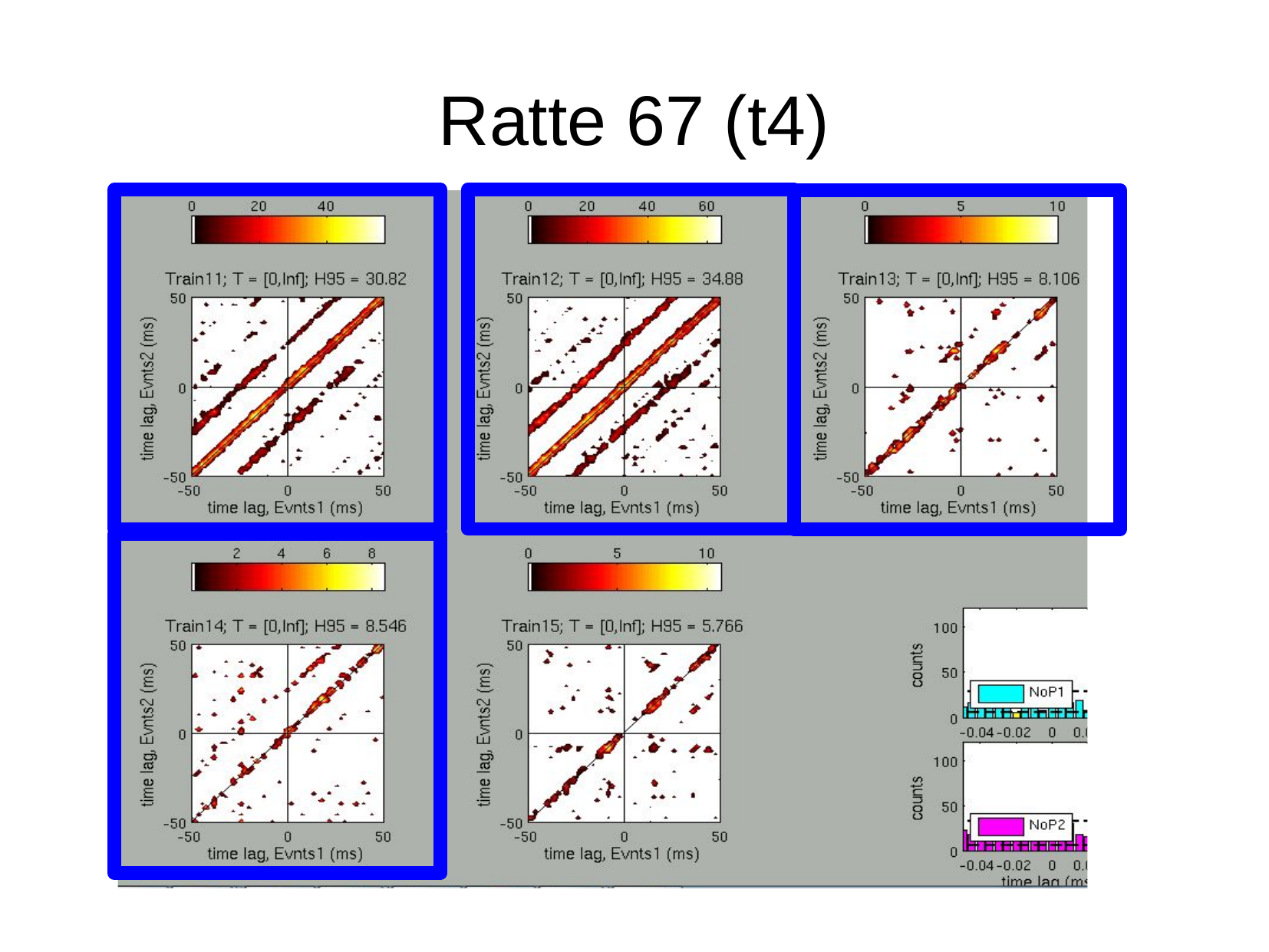

Ratte 67 (t4)

## Slide 27
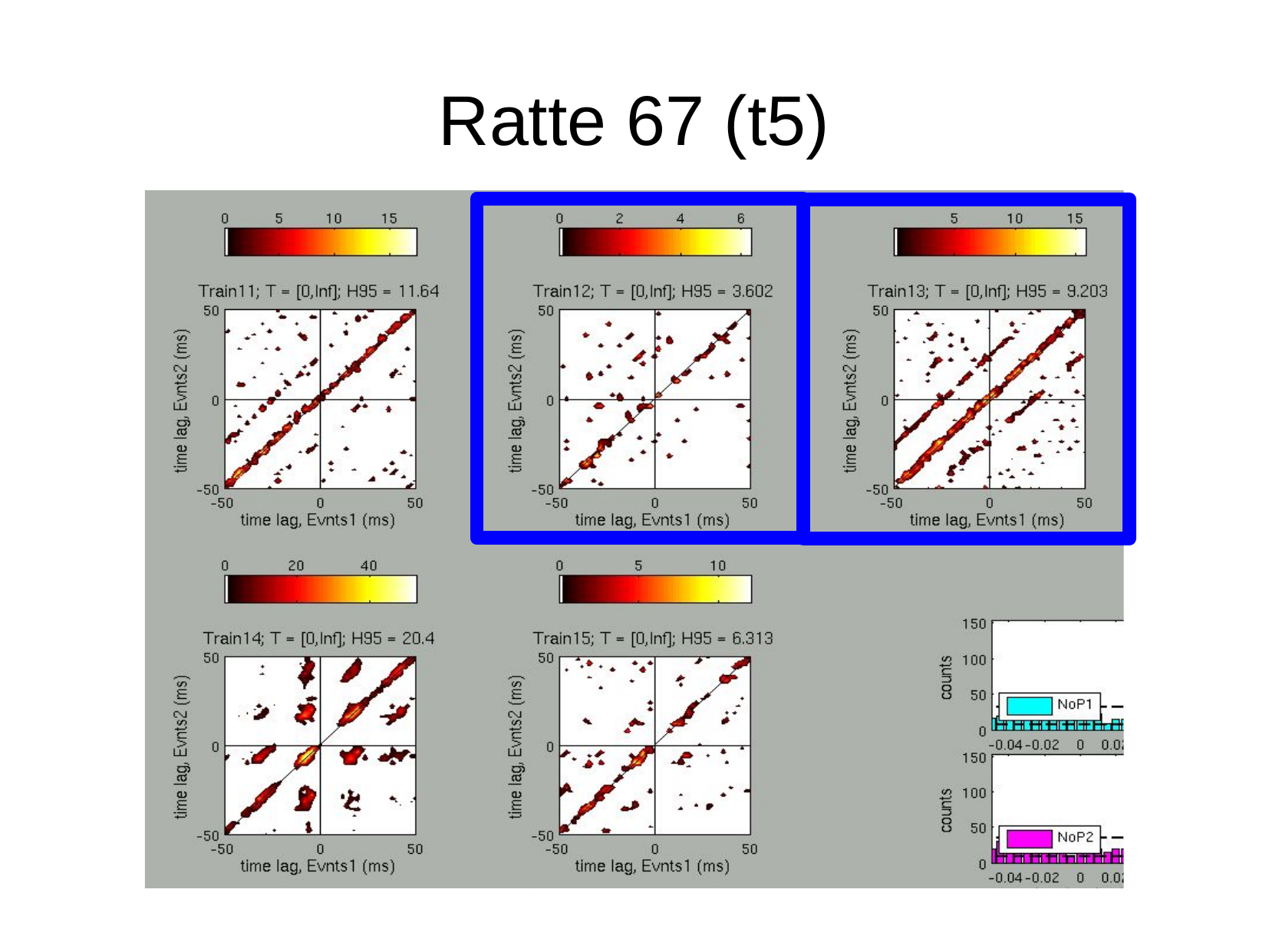

Ratte 67 (t5)

## Slide 28
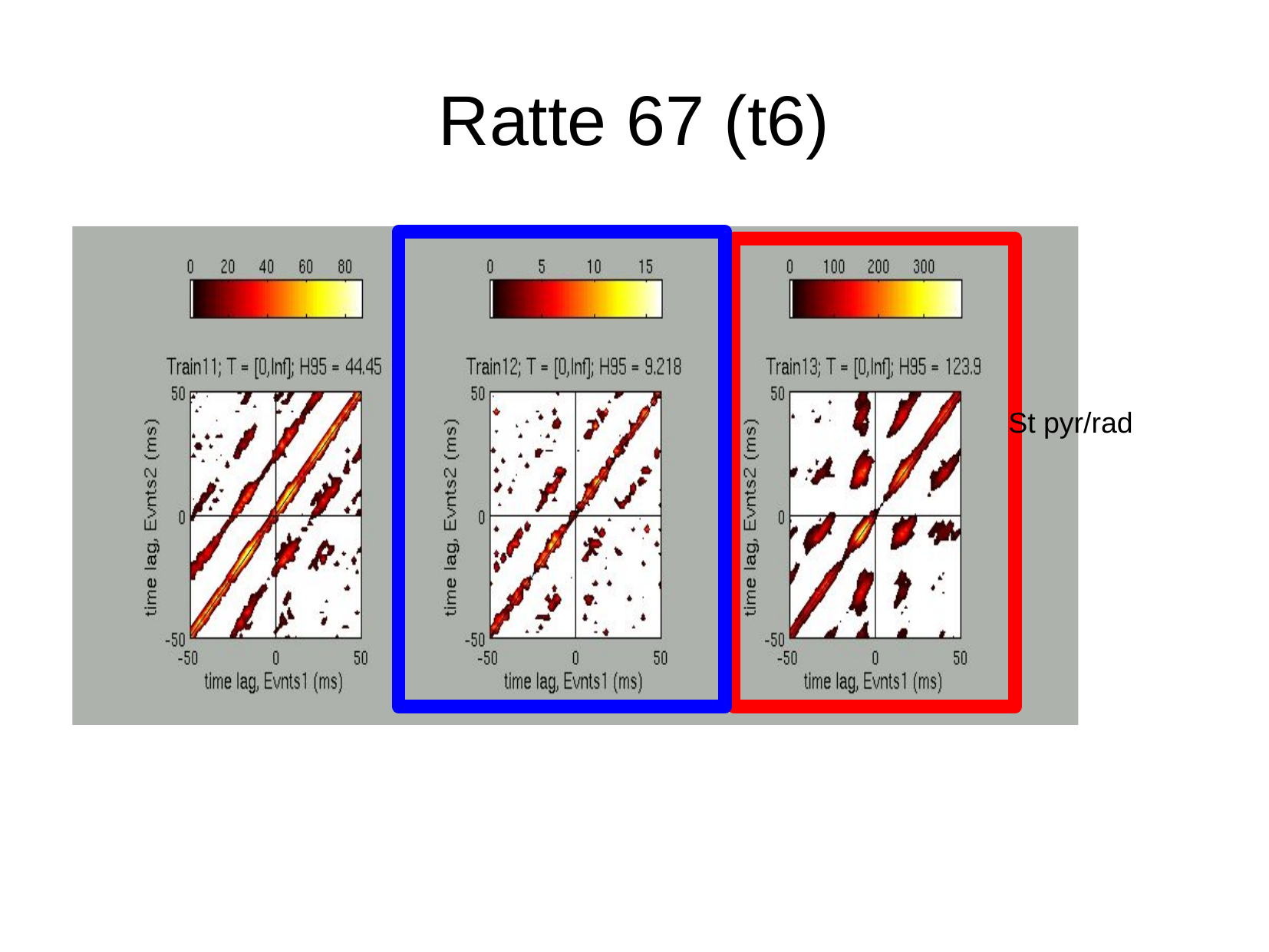

Ratte 67 (t6)
St pyr/rad

## Slide 29
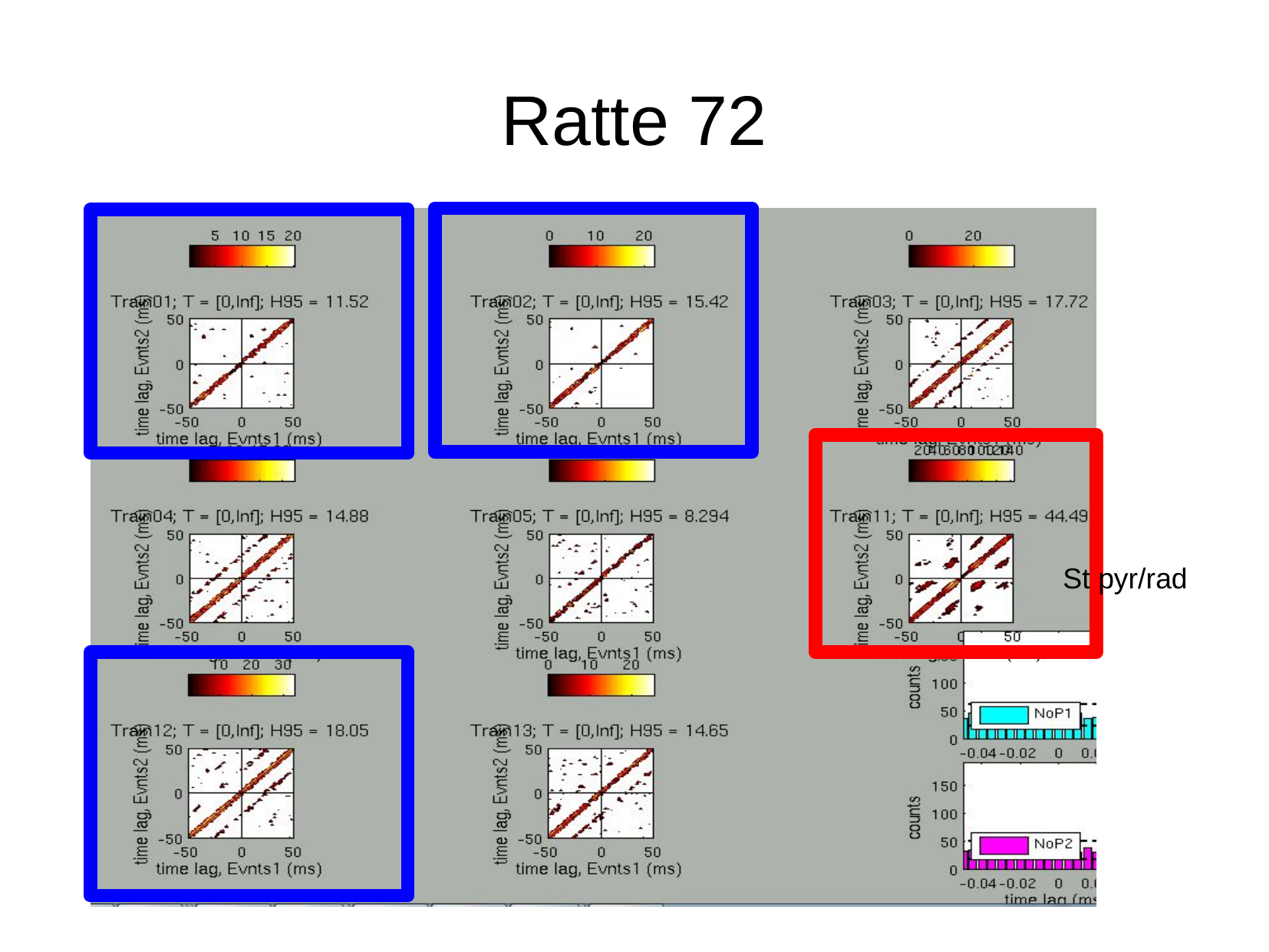

Ratte 72
St pyr/rad
